# Supplementary figures and images for: Detecting the Community Structure and Activity Patterns of Temporal Networks: A Non-Negative Tensor Factorization Approach
Source: PLoS One. 2014 Jan 31;9(1):e86028. doi: 10.1371/journal.pone.0086028 (PMC3908891; doi:10.1371/journal.pone.0086028)

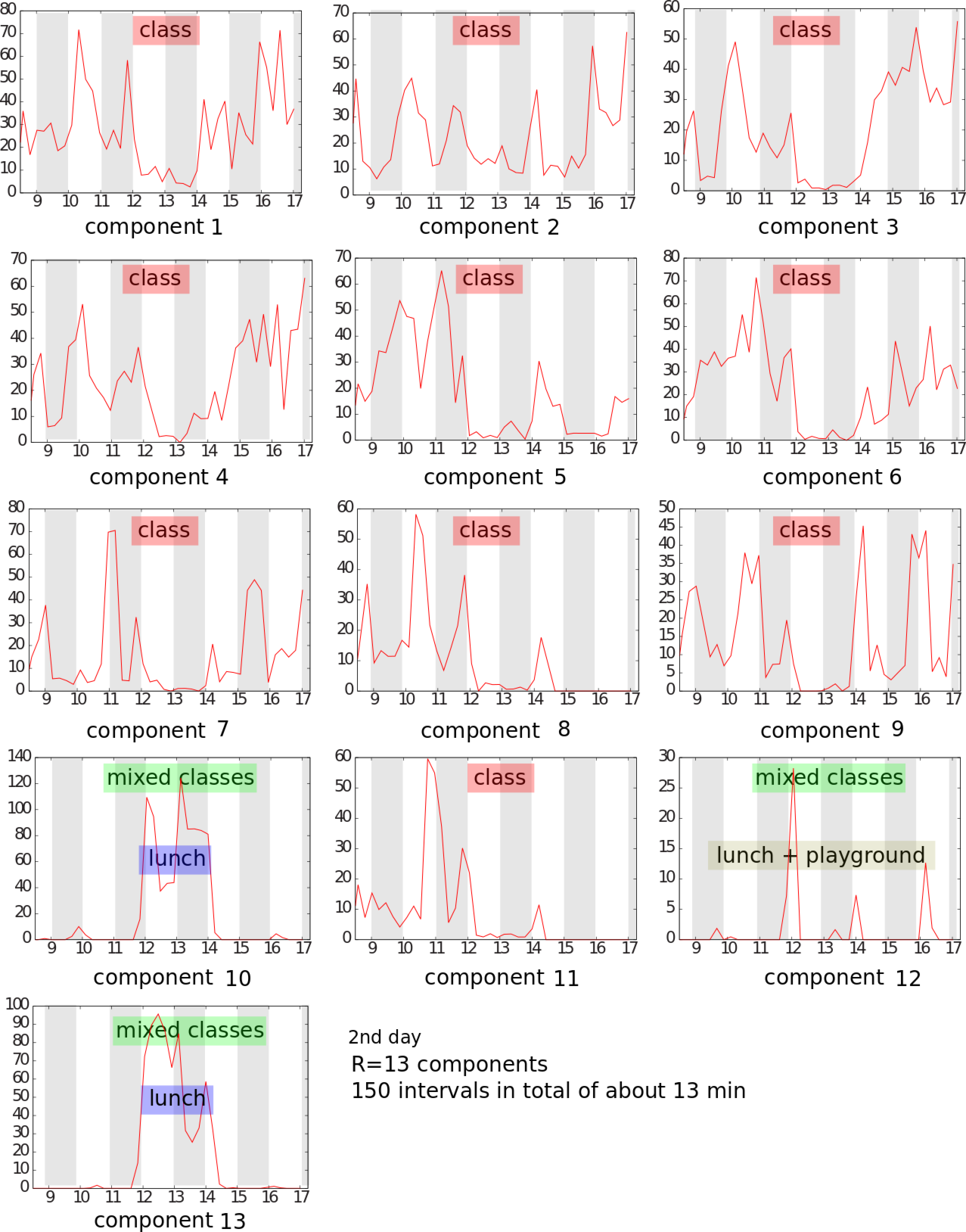

Supplement: Figure S1 — Activity patterns of the extracted components, second day. Each panel corresponds to one component obtained by non-negative tensor factorization of the school temporal network, with , and provides the activity level of the component as a function of the time of the day. Components that can be matched to classes are marked as class. The other three components that correspond to mixed classes exhibit activity patterns that can be understood in terms of gatherings in the social spaces of the school. (TIFF) [file pone.0086028.s001.tiff]

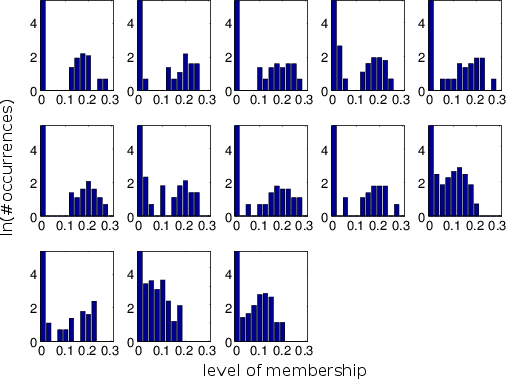

Supplement: Figure S3 — Histograms of membership weights for components. For all components, a large fraction of nodes have zero weights. (TIFF) [file pone.0086028.s003.tiff]

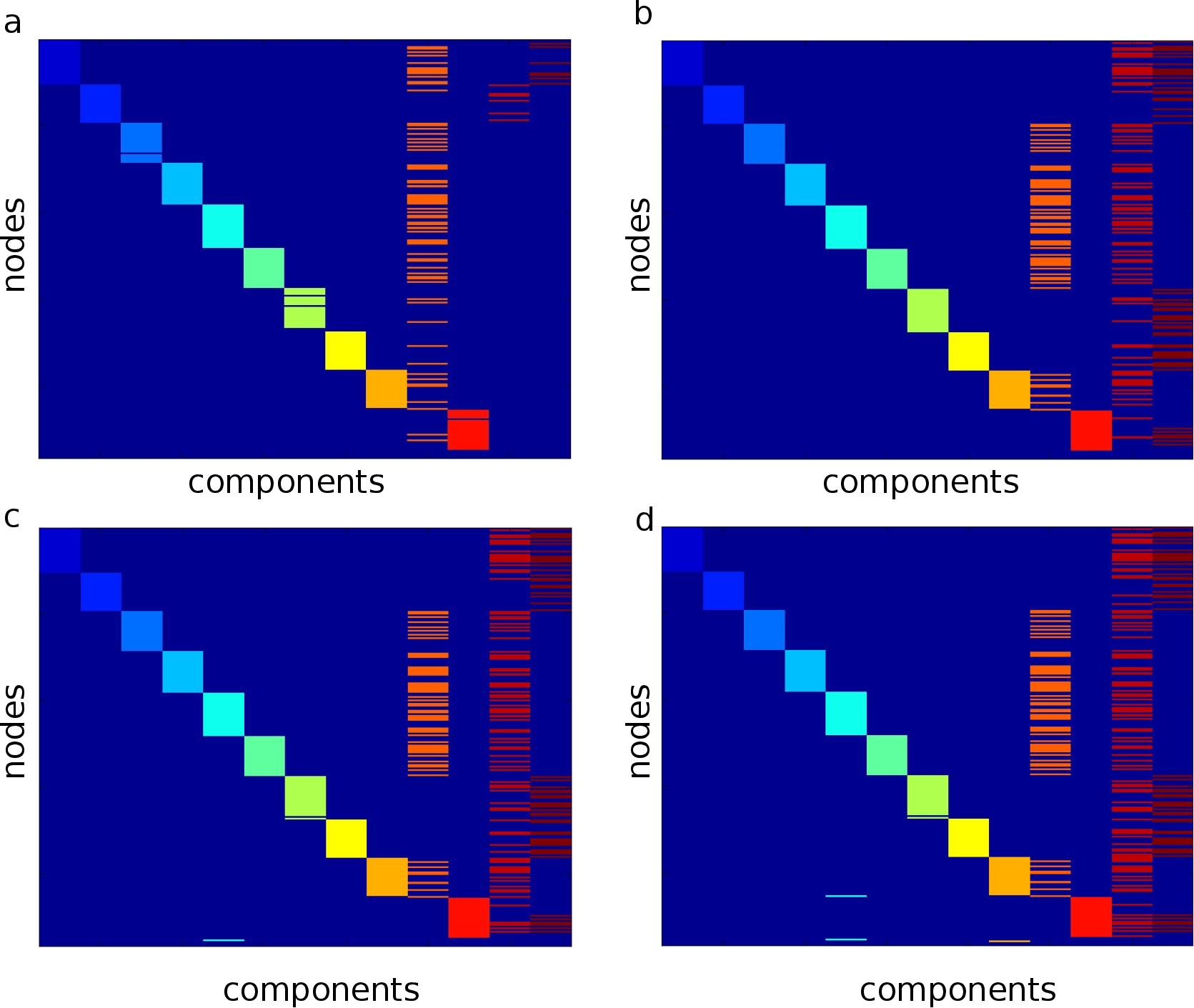

Supplement: Figure S5 — Component-node matrix for components, for different granularities. a) min, b) min, c) min and d) min. Rows correspond to network nodes and columns to components. The matrix is obtained from the factor by classifying each node as belonging (lighter rectangles) or not belonging (dark blue rectangles) to a given component. The order of the nodes has been rearranged to expose the block structure of the matrix. Colors identify components, and the community structures that can be matched to school classes are annotated with the corresponding class name. This figure shows that the general structure of the factor matrices we obtain is very similar: for all values of the interval duration all the school classes are found. (TIFF) [file pone.0086028.s005.tiff]
